# Longitudinal Digital Phenotyping of Traditional Chinese Medicine Constitution From Routine Health Examination Records: Retrospective Clinical Informatics Study

**DOI:** 10.2196/100063

**Published:** 2026-09-23

**Authors:** Yuzhi Huo, Gao Deng, Li Kang, Haizhi Qin, Li Xiao, Ying Deng, Xin Chen, Dan Ding, Fei Wang, Mei Zhang, Min Chen

**Affiliations:** 1 Department of Traditional Chinese Medicine, Chengdu Third People's Hospital, Third People's Hospital of Chengdu, No. 82, Qinglong Street, Chengdu City, Sichuan Province, Chengdu, Sichuan, 610014, China, 1 16602838141; 2 Department of Nuclear Medicine, Affiliated Hospital of Chengdu University of Traditional Chinese Medicine, Chengdu, Sichuan, China; 3 School of Management, Southwestern University of Finance and Economics, Chengdu, Sichuan, China; 4 Chengdu University of Traditional Chinese Medicine, Chengdu, Sichuan, China

**Keywords:** clinical informatics, digital phenotyping, traditional Chinese medicine constitution, routine health examination, electronic health records, machine learning, explainable artificial intelligence, longitudinal cohort, preventive medicine, cardiometabolic risk

## Abstract

**Background:**

Traditional Chinese medicine (TCM) constitution is a structured health state taxonomy used in preventive care, but its relationship with routinely collected health examination data, disease-related markers, and longitudinal change remains difficult to interpret in clinical informatics settings.

**Objective:**

This study aimed to develop and evaluate a longitudinal clinical informatics framework for characterizing TCM constitution as a computable, explainable, record-based phenotype using routine health examination records.

**Methods:**

We conducted a retrospective longitudinal analysis of 47,417 examination-constitution records from 11,355 older adults examined between 2017 and 2026. Baseline analyses used the first available record per participant, and longitudinal analyses used 32,648 pairs of adjacent annual visits. The framework included bidirectional disease-constitution mapping, nonoverlapping multimarker burden modeling, lagged next-visit association models, constitution-state transition analysis, and temporal evaluation of routine examination–based label prediction models. Additional sensitivity analyses evaluated participant overlap across calendar-year splits, participant-disjoint temporal evaluation, BMI-only and BMI-plus-waist baselines, exclusion of anthropometric predictors, adiposity adjustment of longitudinal models, and new-onset and persistence outcomes.

**Results:**

Phlegm-dampness showed the clearest record-based signature and was associated with higher cardiometabolic marker burden than balanced constitution (incidence rate ratio 1.65, 95% CI 1.60‐1.70). In lagged models, its associations with a subsequent abdominal ultrasound abnormality flag and cardiometabolic risk clustering persisted after BMI adjustment, whereas associations with diabetes-related markers, proteinuria, dyslipidemia, and glucose abnormalities were substantially attenuated. Of 11,355 participants, 8122 appeared in at least 2 original calendar-year splits. In a participant-disjoint temporal sensitivity analysis with 1054 new test participants, extreme gradient boosting (XGBoost) identified phlegm-dampness label presence with an area under the receiver operating characteristic curve of 0.927 (95% CI 0.912‐0.941). A BMI-only model achieved 0.923 (95% CI 0.907‐0.938), whereas XGBoost without anthropometric predictors achieved 0.685 (95% CI 0.653‐0.717).

**Conclusions:**

Routine health examination data captured a reproducible, predominantly adiposity-centered phlegm-dampness label phenotype in this cohort of older adults. High discrimination was retained in participant-disjoint evaluation but was nearly matched by the BMI-only model, and several longitudinal associations were explained by adiposity. The models should therefore be interpreted as decision-support and communication aids for an existing constitution assessment process and not as stand-alone diagnostic systems or comprehensive classifiers of TCM constitution.

## Introduction

Clinical informatics increasingly depends on the ability to reuse routinely collected health data to construct interpretable phenotypes for prevention, risk stratification, decision support, and patient-centered communication. Electronic health record phenotyping has made substantial progress in deriving computable disease definitions from structured and unstructured clinical data [1]. Informatics research has also emphasized the need for next-generation phenotype representations that can handle longitudinal, heterogeneous, and multidimensional clinical data [2]. Transportable phenotype algorithms and shared phenotype knowledge bases further illustrate how clinical concepts can be made reproducible across settings [3].

The challenge is broader than conventional disease phenotyping. Health systems often contain locally meaningful categories, patient-reported patterns, functional taxonomies, and culturally embedded health state classifications that are not fully represented by diagnosis codes. Digital phenotyping was initially popularized as moment-by-moment quantification of individual-level human phenotype using data from personal digital devices [4]. Subsequent work has emphasized purpose, quality, safety, and interpretability rather than data volume alone [5]. In this study, we used digital phenotyping in the narrower clinical informatics sense of computationally characterizing an existing health state label from longitudinal routine records; we do not imply continuous or passive behavioral sensing. We used label prediction for models that reproduce an existing recorded constitution label and longitudinal association for models relating current constitution to subsequent recorded marker status. Neither term denotes a stand-alone diagnosis.

Traditional Chinese medicine (TCM) constitution is one such structured health state taxonomy. The constitution framework has been discussed as a basis for individualized and preventive health management in Chinese medicine [6]. Constitution assessment is widely used in China to describe relatively stable yet modifiable patterns of physiological and functional status, commonly grouped into balanced constitution and several biased constitution types, including phlegm-dampness, yang-deficiency, qi-deficiency, and yin-deficiency. The Constitution in Chinese Medicine Questionnaire has been psychometrically validated in Chinese-speaking populations [7], and short-form and Cantonese versions have been evaluated for health management and older adult populations [8,9].

Previous clinical and epidemiological studies have linked constitution patterns, particularly phlegm-dampness, with metabolic syndrome, cardiometabolic risk, and diabetes-related conditions [10,11]. However, much of this evidence remains cross-sectional, condition specific, or focused on conventional association testing. Two practical clinical informatics questions remain insufficiently answered. First, among people with recorded disease histories or examination abnormalities, which constitution patterns are overrepresented? Second, among people with a given constitution pattern, which disease-related markers are currently present or subsequently recorded? These questions matter because patients and clinicians often need bidirectional explanations: what a disease or marker may imply about constitution, and what a constitution label may imply about prevention and follow-up.

Therefore, the relevance of TCM constitution in clinical informatics is not limited to traditional medicine. Constitution can be viewed as a structured health state taxonomy that captures patient-level heterogeneity through a culturally specific lens. If such a taxonomy can be mapped, tracked, and predicted using routine examination data, it may provide a model for studying other non-Western, patient-reported, or locally embedded health classifications. This requires moving beyond single-marker correlation analysis. A computable constitution phenotype should have measurable clinical content, longitudinal behavior, temporal generalizability, model interpretability, and transparent uncertainty. It should also be evaluated using more than discrimination metrics alone because a high area under the receiver operating characteristic curve (AUC) does not guarantee calibration [12] and apparent discrimination does not by itself establish clinical usefulness [13].

In this study, we developed a longitudinal clinical informatics framework for TCM constitution using routine health examination records of older adults. We treated constitution as a structured health state phenotype rather than as a clinical diagnosis. The framework combines bidirectional disease-constitution mapping, multimarker burden modeling, lagged longitudinal association modeling, constitution state transition analysis, and temporally evaluated label prediction from routine examination variables. To align model evaluation with clinical informatics expectations, we incorporated explainable machine learning, calibration assessment, decision curve analysis, sensitivity analysis for unmeasured confounding, and exploratory uncertainty quantification. Our aim was not to deploy an autonomous diagnostic model but to test whether routine examination data can characterize a culturally embedded health taxonomy as an interpretable, reproducible, and longitudinally informative record-based phenotype.

## Methods

### Study Design and Data Source

We conducted a retrospective longitudinal analysis of routine health examination records linked with TCM constitution assessments. The records were collected from residents of 2 communities in Deyang City who underwent routine health examinations between February 22, 2017, and March 30, 2026. The baseline analytic cohort had an observed age range of 64 to 104 years. Each record included demographic information, anthropometric measurements, blood pressure, routine biochemical and hematologic measurements, urine test results, selected examination findings, recorded disease history fields, and TCM constitution labels. These records arose from routine community health management rather than protocol-driven research recruitment.

The analysis was designed to address 3 complementary questions. First, we mapped how recorded diseases and examination-derived disease-related markers were distributed across constitution patterns. Second, we assessed whether baseline and current constitution states were associated with concurrent and next-visit disease or disease marker burden. Third, we evaluated whether routine health examination variables could be used to predict constitution states without using the TCM constitution questionnaire items.

The study size was determined by all eligible routine examination–constitution records available in the source database during the study period; no a priori sample size calculation was performed because the study was retrospective and record based. Reporting was informed by the STROBE (Strengthening the Reporting of Observational Studies in Epidemiology) guidance for observational studies [14] and RECORD (Reporting of Studies Conducted Using Observational Routinely Collected Health Data) guidance for studies using routinely collected health data [15].

### Ethical Considerations

The study used routinely collected health examination and TCM constitution assessment records. The study protocol was reviewed and approved by the Medical Ethics Committee of the Affiliated Hospital of Chengdu University of Traditional Chinese Medicine (approval 2021KL-055). Written informed consent was obtained from participants before inclusion in the routine health examination and constitution assessment database. Analyses were conducted on deidentified records. Publicly released outputs were restricted to analysis code, data dictionaries, aggregate tables, figures, and synthetic demonstration data; participant-level records were not released because they contain sensitive health information.

### Data Cleaning and Variable Harmonization

Raw variables were harmonized into a reproducible analytic data frame. Examination dates were parsed as calendar dates, and examination year was derived from the examination date. Numeric values were extracted from raw fields, and values outside prespecified plausible ranges were set to missing. Bilateral blood pressure measurements were summarized as mean systolic and diastolic blood pressure for continuous analyses and as maximum systolic and diastolic blood pressure for high blood pressure classification. Estimated glomerular filtration rate (eGFR) was calculated from serum creatinine, age, and sex using the 2021 Chronic Kidney Disease Epidemiology Collaboration creatinine equation.

For repeated record analyses, all records with a valid participant identifier, examination date, and constitution label were retained. For baseline descriptive analyses, the first available record per participant was used. For longitudinal analyses, adjacent records for the same participant were paired if the interval between examinations was 180 to 730 days, creating an adjacent annual visit design.

For regression-based association analyses, records were included when the outcome, constitution exposure, and required covariates for the relevant model were nonmissing. For prediction models, missing continuous predictors were imputed using the median within the training pipeline, and missing categorical predictors were encoded as a separate missing category.

### TCM Constitution Assessment and Grouping

Constitution was assessed using the national 33-item Older Adult TCM Constitution Scale, which was incorporated into China’s basic public health service specifications for primary care–based health management of older adults in 2013 [16]. Each item is scored from 1 to 5. Each of the 8 biased constitution types is represented by 4 items: a summed score of 11 or higher indicates that constitution, a score of 9 to 10 indicates a tendency toward it, and a score of 8 or lower indicates its absence. Balanced constitution is assessed using 5 items, of which 4 are reverse scored. A balanced constitution summed score of 17 or higher together with scores of 8 or lower for all 8 biased types indicates balanced constitution. When this definite criterion is not met, the same balanced constitution summed score together with scores of 10 or lower for all 8 biased types indicates a tendency toward balanced constitution. Other score combinations do not meet the balanced constitution criterion. Notably, the standardized phlegm-dampness subscale includes an item scored from BMI categories and an item scored from waist circumference categories.

The routine assessment system automatically applied the standardized rules to calculate constitution scores and generate preliminary classifications. A clinician then reviewed and confirmed the resulting constitution labels. The analytic export retained responses to the 33 items, separate determination fields for all 9 constitution types, an original constitution label field, and a corrected constitution label field. It did not retain assessor identity or a detailed audit trail showing whether and how preliminary classifications changed during clinical confirmation. We therefore analyzed the clinician-confirmed corrected labels recorded in routine practice rather than rerunning the scoring algorithm from individual items.

The recorded labels included balanced constitution (Pinghe), qi-deficiency, yang-deficiency, yin-deficiency, phlegm-dampness, dampness-heat, blood stasis, qi-stagnation, and special diathesis. Multiple labels could be stored in one record. For mutually exclusive descriptive and association analyses, the first-listed label was used as a reproducible primary label convention; the source export did not document whether the first position represented the highest score, clinical dominance, or procedural priority. Primary labels were grouped into balanced, phlegm-dampness, yang-deficiency, qi-deficiency, yin-deficiency, and other biased constitutions. The category other biased constitutions combined dampness-heat, blood stasis, qi-stagnation, and special diathesis because of sparse counts and was interpreted descriptively only.

Balanced constitution was used as the reference group in constitution-to-marker and longitudinal association models. For label prediction, 2 binary targets were defined: any biased primary label vs a balanced primary label and phlegm-dampness appearing anywhere among the recorded labels vs all other records. Thus, the phlegm-dampness prediction target was not restricted to the first-listed label.

### Recorded Diseases and Examination-Derived Risk Markers

Recorded disease history fields were analyzed as recorded diseases rather than adjudicated clinical diagnoses. Disease history fields included cerebrovascular disease, kidney disease, heart disease, vascular disease, eye disease, neurological disease, and other system disease. Examination-derived disease-related markers included electrocardiogram (ECG) abnormality flag, abdominal ultrasound abnormality flag, urine protein trace or positive, urine glucose trace or positive, urine occult blood trace or positive, liver enzyme elevation, anemia, kidney impairment or proteinuria, diabetes-related markers, and cardiometabolic risk clustering. The analytic export contained structured ECG and abdominal ultrasound interpretation codes only; code 1 was harmonized as normal and code 2 as abnormal. It did not retain free-text interpretations or specific abnormalities such as arrhythmia, fatty liver, gallstones, cysts, or masses. These broad fields were therefore treated as heterogeneous screening flags rather than disease diagnoses.

Overweight was defined as BMI ≥24 kg/m^2^ and obesity as BMI ≥28 kg/m^2^. High blood pressure was defined as systolic blood pressure ≥140 mm Hg or diastolic blood pressure ≥90 mm Hg. Fasting glucose thresholds were defined as ≥6.1 mmol/L and ≥7.0 mmol/L. High triglycerides were defined as triglycerides ≥1.7 mmol/L, high total cholesterol as total cholesterol ≥5.2 mmol/L, high low-density lipoprotein cholesterol (LDL-C) as LDL-C ≥3.4 mmol/L, and low high-density lipoprotein cholesterol (HDL-C) as HDL-C <1.0 mmol/L. Any dyslipidemia was defined as any abnormality in triglycerides, total cholesterol, LDL-C, or HDL-C. Kidney impairment or proteinuria was defined as eGFR<60 mL/min/1.73 m^2^ or urine protein trace or positive. Diabetes-related marker was defined as fasting glucose ≥7.0 mmol/L or urine glucose trace or positive. Liver enzyme elevation was defined as alanine aminotransferase (ALT) >40 U/L or aspartate aminotransferase (AST) >40 U/L. Anemia was defined as hemoglobin<130 g/L for men and<120 g/L for women. Cardiometabolic risk clustering was defined as at least 2 positive markers among obesity, high blood pressure, fasting glucose ≥6.1 mmol/L, and any dyslipidemia, requiring at least 2 available component markers.

### Baseline Descriptive Analysis

Baseline characteristics were summarized using the first available record for each participant. Continuous variables were reported as mean (SD), and binary variables were reported as number (percentage). Differences across constitution groups were assessed using Kruskal-Wallis tests for continuous variables and chi-square tests for binary variables. Standardized mean differences vs balanced constitution were calculated to assess the magnitude of between-group imbalance.

### Bidirectional Disease-Constitution Mapping

We performed a bidirectional mapping analysis to answer 2 clinically different questions. The disease-to-constitution analysis estimated, among participants with each recorded disease or examination-derived marker, the proportion belonging to each constitution group. This analysis was intended to answer the patient-facing question: given a disease or marker abnormality, which constitution patterns are most common?

Additionally, we calculated enrichment ratios by comparing the case-specific constitution proportion with the baseline constitution distribution. For example, the phlegm-dampness enrichment ratio for a disease or disease marker was calculated as the percentage of primary phlegm-dampness constitution among cases divided by the percentage of primary phlegm-dampness constitution in the baseline cohort.

The constitution-to-disease analysis addressed the reverse question: given a constitution pattern, which recorded diseases or examination-derived markers are more frequent? For each biased constitution group, age- and sex-adjusted logistic regression models were fitted with balanced constitution as the reference group. Results were reported as odds ratios and 95% CIs. False discovery rate correction was applied across tested associations.

### Constitution-Wide Phenome Association Analysis

To complement the focused disease mapping analyses, we performed a constitution-wide phenome association analysis across routine biochemical, hematologic, anthropometric, and binary risk marker outcomes. For continuous outcomes, values were standardized and modeled using linear regression. For binary outcomes, logistic regression was used. Models compared each biased constitution group with balanced constitution and adjusted for age, sex, and examination year. Because participants could contribute repeated records, robust SEs clustered by participant were used. This analysis was used primarily for heat map visualization and hypothesis generation rather than for causal inference.

### Multimarker Burden Modeling

To summarize overall clinical burden beyond individual binary markers, we constructed 3 marker burden scores at baseline: recorded disease burden, cardiometabolic marker burden, and examination-derived marker burden. For each participant, the number of positive markers was divided by the number of nonmissing eligible markers. The recorded disease burden included recorded cerebrovascular, kidney, heart, vascular, eye, and neurological disease fields. The cardiometabolic marker burden included obesity, high blood pressure, fasting glucose ≥6.1 mmol/L, high triglycerides, high LDL-C, low HDL-C, and eGFR<60 mL/min/1.73 m^2^. In the revised examination-derived burden, all components were mutually nonoverlapping: high blood pressure, fasting glucose ≥7.0 mmol/L, any dyslipidemia, ECG abnormality flag, an abdominal ultrasound abnormality flag, proteinuria, glycosuria, hematuria, liver enzyme elevation, anemia, and eGFR<60 mL/min/1.73 m^2^. The earlier composite component kidney impairment or proteinuria was removed so that proteinuria would not be counted twice.

Constitution-specific burden associations were estimated using robust Poisson regression with the log number of available markers as an offset. Models were adjusted for age and sex. Results were reported as incidence rate ratios relative to balanced constitution.

### Lagged Longitudinal Risk Modeling

For longitudinal analyses, pairs of adjacent annual visits were used to evaluate associations between current constitution and subsequent disease-related marker status. For each current biased constitution group, analyses were restricted to records with either balanced constitution or the target biased constitution at the current visit. Logistic regression models were fitted for next-visit outcome status, with the target constitution indicator as the exposure. Models were adjusted for current status of the same outcome, current age, sex, follow-up interval in years, and current examination year. Robust SEs clustered by participant were used to account for repeated observations. These models estimated subsequent status conditional on current status; they were not interpreted as causal effects or confirmed disease development.

New-onset analyses were restricted to visit pairs in which the marker was normal or absent at the current visit, and persistence analyses were restricted to pairs in which the marker was positive at the current visit. Recurrence was not analyzed because it requires at least 3 appropriately spaced observations and a prespecified remission interval. For the main phlegm-dampness outcomes, additional sensitivity models adjusted for current BMI, current waist circumference, and both measures together to distinguish associations that persisted from those explained by body size. False discovery rate correction was applied across the original longitudinal association tests; the additional adiposity-adjusted models were interpreted as sensitivity analyses.

### Constitution State Transition Analysis

Constitution state dynamics were described using pairs of adjacent annual visits. A transition matrix was constructed by cross-tabulating the current constitution group against the next-visit constitution group. The crude change rate was defined as the proportion of adjacent pairs in which the next constitution group differed from the current constitution group.

We then modeled the predictors of 2 clinically interpretable transitions: transition from balanced constitution to any biased constitution and transition from phlegm-dampness constitution to balanced constitution. Candidate predictors included age, BMI, waist circumference, systolic blood pressure, diastolic blood pressure, fasting glucose, triglycerides, HDL-C, LDL-C, ALT, creatinine, eGFR, and hemoglobin. Continuous predictors were standardized, and logistic regression models estimated ORs per SD increment. Models were adjusted for age when age was not the predictor, sex, follow-up interval, and current examination year; robust SEs clustered by participant were used.

### Temporal Validation of Digital Constitution Prediction Models

We developed routine examination–based prediction models for 2 constitution screening tasks: any biased constitution vs balanced constitution and phlegm-dampness label presence vs all other records. TCM questionnaire items were not included as predictors. Candidate predictors included age, height, weight, waist circumference, BMI, mean systolic and diastolic blood pressure, hemoglobin, white blood cell count, platelet count, fasting glucose, ALT, AST, total bilirubin, creatinine, urea, eGFR, total cholesterol, triglycerides, LDL-C, HDL-C, lifestyle variables, self-rated health and function fields, cognitive and emotional assessment fields, and recorded disease history fields.

The original calendar-year evaluation used 2017‐2023 records for model training, 2024 records for threshold selection, and 2025‐2026 records for testing. Because participants could contribute records in more than 1 period, this design was interpreted as future record prediction within a recurring examination cohort rather than as independent validation. We quantified participant overlap across all 3 periods. As an additional sensitivity analysis, we then implemented a participant-disjoint temporal evaluation based on each participant’s first entry into the entire cohort: participants first observed in 2017‐2023 were assigned to training, those first observed in 2024 to validation, and those first observed in 2025‐2026 to testing. The first eligible labeled record per participant was used within each disjoint split, and no participant appeared in more than 1 split.

The evaluation was aligned with the Transparent Reporting of a Multivariable Prediction Model for Individual Prognosis or Diagnosis (TRIPOD) principles [17] and the updated TRIPOD + AI guidance [18]. Two modeling strategies were compared: penalized logistic regression and XGBoost. Classification thresholds were selected in the validation set by maximizing balanced accuracy and applied unchanged to the test set. We additionally fitted BMI-only and BMI-plus-waist logistic regression baselines and repeated the full models after removing height, weight, waist circumference, and BMI. Test set AUC, precision-recall AUC (PR-AUC), and Brier score were accompanied by 95% percentile bootstrap CIs in the participant-disjoint analysis.

Model discrimination and classification performance were evaluated using AUC, average precision (PR-AUC), balanced accuracy, *F*_1_-score, precision, recall, specificity, confusion matrix counts, and Brier score. The evaluation strategy was designed to reduce avoidable prediction model bias and applicability concerns emphasized in Prediction Model Risk of Bias Assessment Tool (PROBAST) + AI [19]. As a sensitivity analysis, all anthropometric variables directly related to body size and adiposity (height, weight, waist circumference, and BMI) were removed, and models were refit using the same temporal validation framework.

### Explainability, Calibration, Decision Curve Analysis, and Uncertainty Quantification

For the XGBoost models, Shapley additive explanations (SHAP) analysis was performed on a sample of temporal test records to estimate the mean absolute contribution of each predictor to model output [20]. Feature importance was summarized using mean absolute SHAP values.

Calibration was assessed in the temporal test set using deciles of predicted probability, Brier score, calibration intercept, calibration slope, 10-bin expected calibration error, and integrated absolute calibration error. Decision curve analysis was performed across prespecified threshold probabilities to estimate model net benefit relative to treat-all and treat-none strategies.

As exploratory uncertainty quantification, split conformal set prediction was applied to XGBoost model probabilities using the 2024 validation set as the calibration set and the 2025‐2026 temporal test set as the evaluation set. Conformal prediction provides a distribution-free framework for prediction sets under exchangeability assumptions [21]. Clinical applications still require caution because temporal shift can undermine nominal coverage [22]. Because the temporal test period showed a distribution shift, conformal results were interpreted as temporal stress test summaries rather than as guaranteed clinical coverage estimates.

### Quantitative Sensitivity and Subgroup Analyses

For selected longitudinal associations, E-values were calculated to quantify the minimum strength of association that an unmeasured confounder would need to have with both the constitution exposure and next-visit outcome, conditional on measured covariates, to explain away the observed association [23]. E-values were reported for the point estimate and for the confidence limit closest to the null.

The main phlegm-dampness associations were repeated by sex and by age group using the cohort median age as the cut point. Interaction terms were tested to evaluate whether the association between the phlegm-dampness constitution and key outcomes differed by sex or age group. Medication names were present in 42.3% of records, but a blank field could not be distinguished from no medication use, and drug names were not standardized for the source database. Medication was therefore not included as a covariate; its incomplete ascertainment was treated as a source of residual confounding.

### Reproducibility

All data cleaning, statistical analyses, prediction modeling, and figure generation were implemented using reproducible scripts. Main analysis outputs were saved as structured CSV tables, and publication figures were exported as vector files (PDF/scalable vector graphics [SVG]) and high-resolution bitmap files (PNG/TIFF). The raw Microsoft Excel workbook was not modified during analysis. For public reproducibility, the repository package contains executable scripts, environment specifications, a file manifest, data dictionaries, aggregate result tables, submission-oriented figure files, and synthetic demonstration data that reproduce the expected feature schema without exposing participant-level records.

## Results

### Study Population and Digital Phenotyping Framework

The source workbook contained 47,417 examination-constitution records from 11,355 unique older adults between February 22, 2017, and March 30, 2026. Constitution labels were available for 47,411 records, with only 6 records missing constitution information. The median number of records per participant was 4 (IQR 2-6, maximum 11), and 9134 participants had at least 2 records.

**Figure 1.**
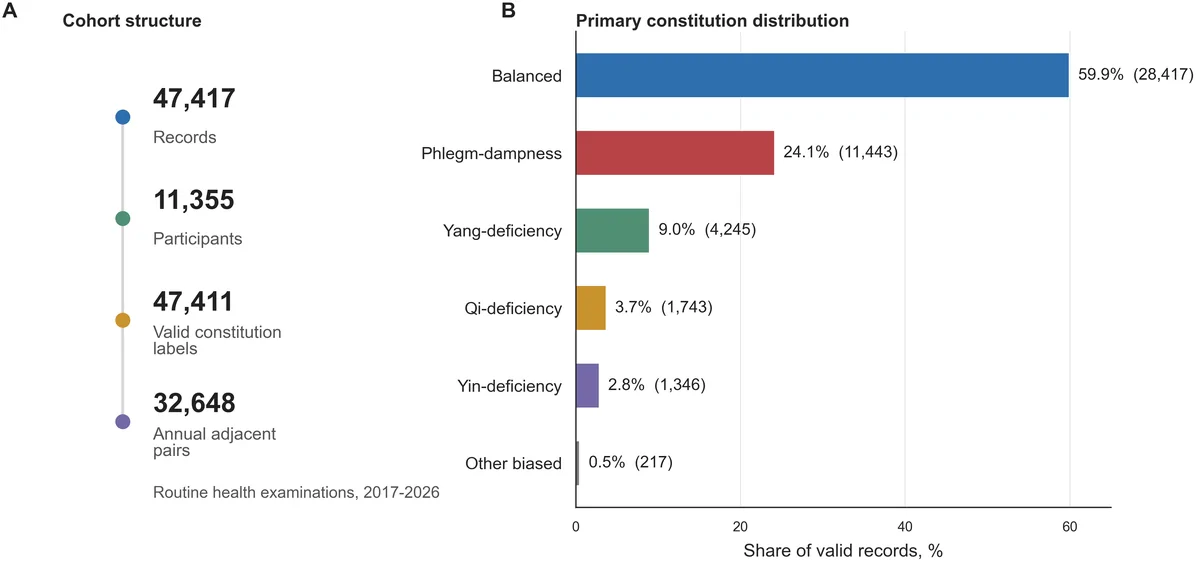
Study cohort and constitution distribution. (A) Overview of the routine health examination cohort; (B) the distribution of primary traditional Chinese medicine constitution groups.

Across all valid records, balanced constitution (Pinghe) accounted for 28,417 of 47,417 records (59.9%), followed by phlegm-dampness constitution (11,443/47,417; 24.1%), yang-deficiency constitution (4,245/47,417; 9.0%), qi-deficiency constitution (1,743/47,417; 3.7%), yin-deficiency constitution (1,346/47,417; 2.8%), and other biased constitutions (217/47,417; 0.5%). The baseline descriptive analysis used the first available record for each participant (n=11,354). In this baseline cohort, 7786 participants had balanced constitution, 2215 had phlegm-dampness constitution, 699 had yang-deficiency constitution, 316 had qi-deficiency constitution, 311 had yin-deficiency constitution, and 27 had other biased constitutions. Because the other biased constitutions group was extremely small, its estimates were treated as descriptive only. Annual lagged analyses included 32,648 adjacent record pairs with an interval of 180 to 730 days; the median follow-up interval was 363 (IQR 348‐383) days (Figure 1).

The overall analytic framework treated TCM constitution as a structured health state phenotype rather than as a diagnosis. We used routine examination data to construct bidirectional disease-constitution maps, multimarker burden models, subsequent status association models, transition models, and temporally evaluated label prediction models.

### Baseline Characteristics by Constitution Group

In the baseline cohort, phlegm-dampness constitution showed a distinct adiposity-centered cardiometabolic profile (Table 1). Mean BMI was 28.34 (SD 2.59) in the phlegm-dampness constitution group compared with 23.65 (SD 2.74) in the balanced constitution group, and mean waist circumference was 94.02 (SD 6.79) vs 81.01 (SD 7.04) cm. Overweight was present in 2175 of 2215 (98.2%) participants with phlegm-dampness constitution compared with 3473 of 7784 (44.6%) participants with balanced constitution; the corresponding proportions for obesity were 49.9% (1106/2215) and 5.3% (410/7784) for participants with phlegm-dampness constitution and balanced constitution, respectively.

**Table 1.** Baseline characteristics by constitution group.^a^

Characteristic	Balanced	Phlegm-dampness	Yang-deficiency	Qi-deficiency	Yin-deficiency	Other biased	Overall	*P* value	Maximum SMD^b^
Number of observations (N)	7786	2215	699	316	311	27	11,354	—^c^	—
Age (years), mean (SD)	75.36 (6.41); n=7786	73.38 (5.84); n=2215	75.02 (7.36); n=699	78.18 (7.64); n=316	75.52 (6.89); n=311	73.04 (6.27); n=27	75.03 (6.48); n=11,354	<.001	0.4
BMI (kg/m^2^), mean (SD)	23.65 (2.74); n=7784	28.34 (2.59); n=2215	22.99 (3.22); n=699	24.11 (3.78); n=316	24.14 (3.18); n=311	23.06 (2.28); n=27	24.55 (3.36); n=11,352	<.001	1.76
Waist circumference (cm), mean (SD)	81.01 (7.04); n=7784	94.02 (6.79); n=2215	80.89 (9.02); n=699	83.97 (9.67); n=316	83.63 (8.63); n=311	81.74 (5.79); n=27	83.70 (8.88); n=11,352	<.001	1.88
Systolic BP^d^, (mm Hg), mean (SD)	140.02 (18.60); n=7785	143.06 (17.53); n=2215	136.38 (18.31); n=699	137.61 (17.32); n=316	140.02 (17.49); n=311	138.89 (16.81); n=27	140.32 (18.38); n=11,353	<.001	0.2
Diastolic BP (mm Hg), mean (SD)	83.20 (10.67); n=7785	85.57 (10.35); n=2215	81.48 (10.00); n=699	81.78 (10.08); n=316	82.98 (9.78); n=311	84.33 (11.43); n=27	83.52 (10.58); n=11,353	<.001	0.22
Fasting glucose (mmol/L), mean (SD)	5.94 (2.09); n=7758	6.23 (2.05); n=2208	5.68 (1.95); n=695	6.36 (2.50); n=314	6.42 (2.44); n=310	5.60 (1.57); n=27	6.01 (2.10); n=11,312	<.001	0.21
Triglycerides (mmol/L), mean (SD)	1.63 (1.53); n=7752	1.97 (1.56); n=2201	1.49 (1.03); n=694	1.60 (1.37); n=316	1.77 (1.36); n=306	1.95 (2.59); n=27	1.69 (1.51); n=11,296	<.001	0.22
HDL-C^e^ (mmol/L), mean (SD)	1.65 (0.43); n=6566	1.50 (0.36); n=2111	1.68 (0.44); n=644	1.64 (0.43); n=249	1.52 (0.41); n=261	1.55 (0.37); n=24	1.62 (0.42); n=9855	<.001	0.4
LDL-C^f^ (mmol/L), mean (SD)	2.48 (0.88); n=6567	2.72 (0.82); n=2110	2.68 (0.87); n=645	2.66 (0.90); n=249	2.51 (0.95); n=261	2.98 (0.85); n=24	2.55 (0.87); n=9856	<.001	0.57
ALT (U/L), mean (SD)	21.22 (14.17); n=7737	25.82 (17.89); n=2204	19.91 (13.98); n=696	22.95 (29.38); n=316	22.43 (15.26); n=305	20.22 (12.59); n=27	22.11 (15.70); n=11,285	<.001	0.29
Creatinine (µmol/L), mean (SD)	69.77 (27.54); n=7746	69.72 (23.01); n=2203	68.12 (24.36); n=694	72.52 (29.32); n=316	72.87 (39.70); n=306	68.70 (22.81); n=27	69.82 (26.99); n=11,292	.015	0.1
Hemoglobin (g/L), mean (SD)	135.92 (13.99); n=7748	139.95 (14.68); n=2202	134.59 (14.61); n=694	135.36 (17.40); n=314	135.62 (13.87); n=306	136.93 (13.70); n=27	136.60 (14.36); n=11,291	<.001	0.28
Female sex, n/N (%)	4296/7786 (55.2%)	1171/2215 (52.9%)	461/699 (66.0%)	182/316 (57.6%)	183/311 (58.8%)	16/27 (59.3%)	6309/11354 (55.6%)	<.001	0.22
Overweight (BMI ≥24 kg/m^2^), n/N (%)	3473/7784 (44.6%)	2175/2215 (98.2%)	249/699 (35.6%)	147/316 (46.5%)	149/311 (47.9%)	8/27 (29.6%)	6201/11352 (54.6%)	<.001	1.47
Obesity (BMI ≥28 kg/m^2^), n/N (%)	410/7784 (5.3%)	1106/2215 (49.9%)	39/699 (5.6%)	46/316 (14.6%)	41/311 (13.2%)	1/27 (3.7%)	1643/11352 (14.5%)	<.001	1.15
High BP (≥140/90 mm Hg), n/N (%)	4001/7785 (51.4%)	1449/2215 (65.4%)	330/699 (47.2%)	154/316 (48.7%)	159/311 (51.1%)	16/27 (59.3%)	6109/11353 (53.8%)	<.001	0.29
FPG^g^ ≥6.1 mmol/L, n/N (%)	1904/7758 (24.5%)	743/2208 (33.7%)	136/695 (19.6%)	107/314 (34.1%)	112/310 (36.1%)	6/27 (22.2%)	3008/11312 (26.6%)	<.001	0.25
FPG ≥7.0 mmol/L, n/N (%)	1147/7758 (14.8%)	421/2208 (19.1%)	75/695 (10.8%)	65/314 (20.7%)	76/310 (24.5%)	2/27 (7.4%)	1786/11312 (15.8%)	<.001	0.25
High triglycerides, n/N (%)	2226/7752 (28.7%)	975/2201 (44.3%)	184/694 (26.5%)	90/316 (28.5%)	111/306 (36.3%)	9/27 (33.3%)	3595/11296 (31.8%)	<.001	0.33
Low HDL-C, n/N (%)	163/6566 (2.5%)	102/2111 (4.8%)	24/644 (3.7%)	12/249 (4.8%)	13/261 (5.0%)	2/24 (8.3%)	316/9855 (3.2%)	<.001	0.26
eGFR^h^ <60 mL/min/1.73 m^2^, n/N (%)	568/7746 (7.3%)	155/2203 (7.0%)	48/694 (6.9%)	40/316 (12.7%)	27/306 (8.8%)	3/27 (11.1%)	841/11292 (7.4%)	.011	0.18
Any dyslipidemia, n/N (%)	4668/7753 (60.2%)	1467/2202 (66.6%)	420/694 (60.5%)	188/316 (59.5%)	192/306 (62.7%)	19/27 (70.4%)	6954/11298 (61.6%)	<.001	0.21

a Continuous variables are presented as mean (SD); n=available observations. Binary variables are presented as events/available observations (%).

b SMD: standardized mean difference.

c Not applicable.

d BP: blood pressure.

e HDL-C: high-density lipoprotein cholesterol.

f LDL-C: low-density lipoprotein cholesterol.

g FPG: fasting plasma glucose.

h eGFR: estimated glomerular filtration rate.

Phlegm-dampness constitution was also characterized by higher cardiometabolic marker burden. High blood pressure was observed in 1449 of 2215 (65.4%) participants in the phlegm-dampness group vs 4001 of 7785 (51.4%) participants in the balanced group. Fasting glucose ≥6.1 mmol/L was present in 743 of 2208 (33.7%) vs 1904 of 7758 (24.5%) participants with the phlegm-dampness constitution and balanced constitution, respectively, and high triglycerides were present in 975 of 2201 (44.3%) vs 2226 of 7752 (28.7%) participants with the phlegm-dampness constitution and balanced constitution, respectively.

### Bidirectional Disease-Constitution Mapping

**Figure 2.**
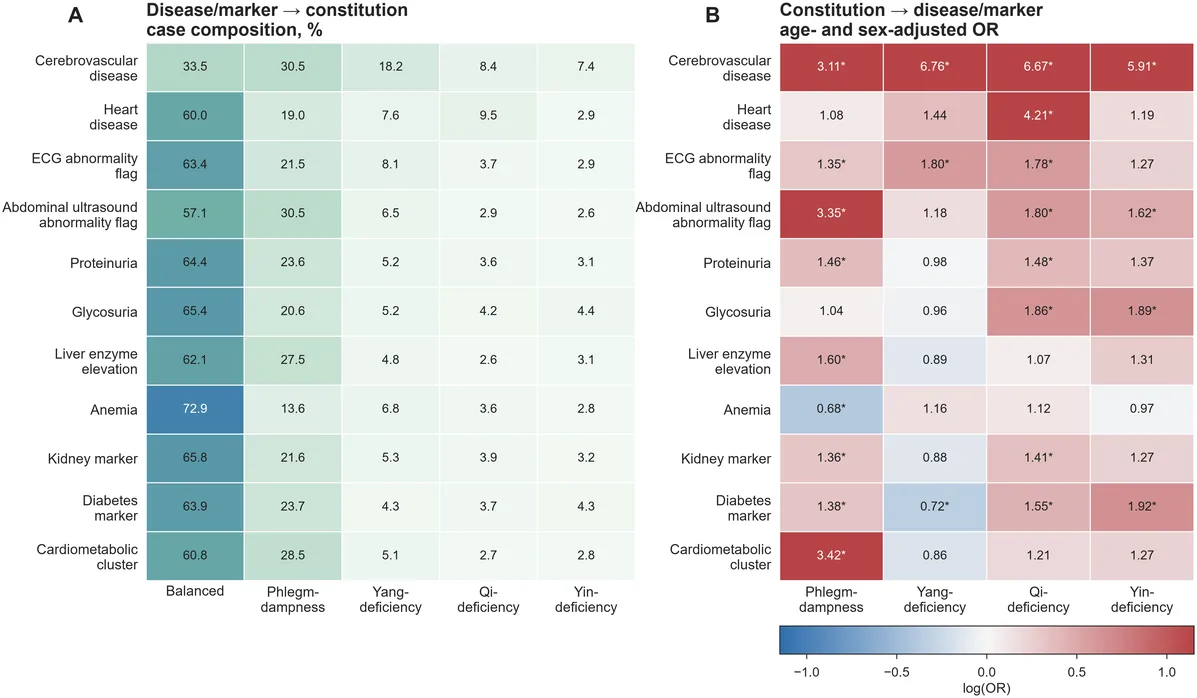
Bidirectional constitution-disease map at baseline. (A) Constitution composition among participants with each recorded disease or examination-derived marker; (B) age- and sex-adjusted odds ratios relative to balanced constitution. Cell labels show percentages or odds ratios; * indicates a false discovery rate of q<0.05. ECG: electrocardiogram; OR: odds ratio.

We first examined the patient-facing question of which constitution patterns were common among participants with recorded diseases or disease-related examination markers. Several disease or disease marker groups showed a higher proportion of biased constitution overall and a higher proportion of primary phlegm-dampness constitution (Figure 2, panel A). Among participants with an abdominal ultrasound abnormality flag, biased constitution accounted for 42.9% (2284/5323), and primary phlegm-dampness constitution accounted for 30.6% (1626/5323) (enrichment ratio vs baseline 1.57). Among participants with recorded cerebrovascular disease, biased constitution accounted for 66.5% (135/203), and primary phlegm-dampness constitution accounted for 30.5% (62/203) (enrichment ratio 1.57). Among participants with cardiometabolic risk clustering, biased constitution accounted for 39.2% (2253/5744), and primary phlegm-dampness constitution accounted for 28.5% (1635/5744) (enrichment ratio 1.46). Primary phlegm-dampness constitution was also enriched among participants with liver enzyme elevation, diabetes-related markers, urine protein trace or positive, kidney impairment or proteinuria, and an ECG abnormality flag.

Next, we examined the reverse question of which recorded diseases or disease-related markers were more common in each constitution group compared with balanced constitution, adjusted for age and sex (Figure 2 panel B; Table 2). Phlegm-dampness constitution was strongly associated with cardiometabolic markers and an abdominal ultrasound abnormality flag. Compared with balanced constitution, phlegm-dampness constitution was associated with cardiometabolic risk clustering (OR 3.42, 95% CI 3.07‐3.80), an abdominal ultrasound abnormality flag (OR 3.35, 95% CI 2.98‐3.76), liver enzyme elevation (OR 1.60, 95% CI 1.38‐1.84), diabetes-related markers (OR 1.38, 95% CI 1.22‐1.56), and kidney impairment or proteinuria (OR 1.36, 95% CI 1.21‐1.53).

**Table 2.** Baseline associations of constitutions with diseases and disease-related markers.^a^

Outcome	Phlegm-dampness	Yang-deficiency	Qi-deficiency	Yin-deficiency
Recorded cerebrovascular disease	3.11 (2.18‐4.42)*	6.76 (4.48‐10.21)*	6.67 (3.84‐11.58)*	5.91 (3.33‐10.47)*
Recorded heart disease	1.08 (0.65‐1.81)	1.44 (0.68‐3.02)	4.21 (2.12‐8.34)*	1.19 (0.37‐3.81)
ECG^b^ abnormality flag	1.35 (1.22‐1.49)*	1.80 (1.54‐2.10)*	1.78 (1.42‐2.24)*	1.27 (1.01‐1.61)
Abdominal ultrasound abnormality flag	3.35 (2.98‐3.76)*	1.18 (1.00‐1.40)	1.80 (1.37‐2.36)*	1.62 (1.22‐2.14)*
Liver enzyme elevation	1.60 (1.38‐1.84)*	0.89 (0.67‐1.18)	1.07 (0.73‐1.58)	1.31 (0.92‐1.88)
Diabetes-related marker	1.38 (1.22‐1.56)*	0.72 (0.56‐0.91)*	1.55 (1.18‐2.04)*	1.92 (1.47‐2.49)*
Kidney impairment or proteinuria	1.36 (1.21‐1.53)*	0.88 (0.71‐1.08)	1.41 (1.09‐1.82)*	1.27 (0.96‐1.67)
Cardiometabolic risk cluster	3.42 (3.07‐3.80)*	0.86 (0.73‐1.00)	1.21 (0.96‐1.52)	1.27 (1.01‐1.59)

a Values are age- and sex-adjusted odds ratios (95% CIs) for each biased constitution group relative to balanced constitution. * indicates a false discovery rate-adjusted q value <0.05.

b ECG: electrocardiogram.

Recorded cerebrovascular disease showed a broader pattern across biased constitutions. Compared with balanced constitution, the odds of recorded cerebrovascular disease were higher for phlegm-dampness constitution (OR 3.11, 95% CI 2.18‐4.42), yang-deficiency constitution (OR 6.76, 95% CI 4.48‐10.21), qi-deficiency constitution (OR 6.67, 95% CI 3.84‐11.58), and yin-deficiency constitution (OR 5.91, 95% CI 3.33‐10.47). An ECG abnormality flag was also more common in phlegm-dampness, yang-deficiency, and qi-deficiency constitutions. Compared with balanced constitution, Yin-deficiency constitution showed a stronger association with diabetes-related markers than balanced constitution (OR 1.92, 95% CI 1.47‐2.49). while qi-deficiency constitution showed higher odds of recorded heart disease (OR 4.21, 95% CI 2.12‐8.34) and kidney impairment or proteinuria (OR 1.41, 95% CI 1.09‐1.82).

### Multimarker Burden Modeling

To move beyond single-marker associations, we summarized recorded disease burden, cardiometabolic marker burden, and examination-derived marker burden. Compared with balanced constitution, phlegm-dampness constitution was associated with a higher cardiometabolic marker burden (incidence rate ratio [IRR] 1.65, 95% CI 1.60‐1.70), corrected nonoverlapping examination–derived marker burden (IRR 1.23, 95% CI 1.20‐1.25), and recorded disease burden (IRR 1.38, 95% CI 1.10‐1.72) after adjustment for age and sex.

Other biased constitutions showed a different burden profile. Recorded disease burden was higher in yang-deficiency constitution (IRR 2.07, 95% CI 1.54‐2.78), qi-deficiency constitution (IRR 2.77, 95% CI 1.93‐4.00), and yin-deficiency constitution (IRR 2.33, 95% CI 1.49‐3.62), whereas the cardiometabolic marker burden was most prominent for phlegm-dampness constitution.

### Longitudinal Constitution-to-Risk Associations

**Figure 3.**
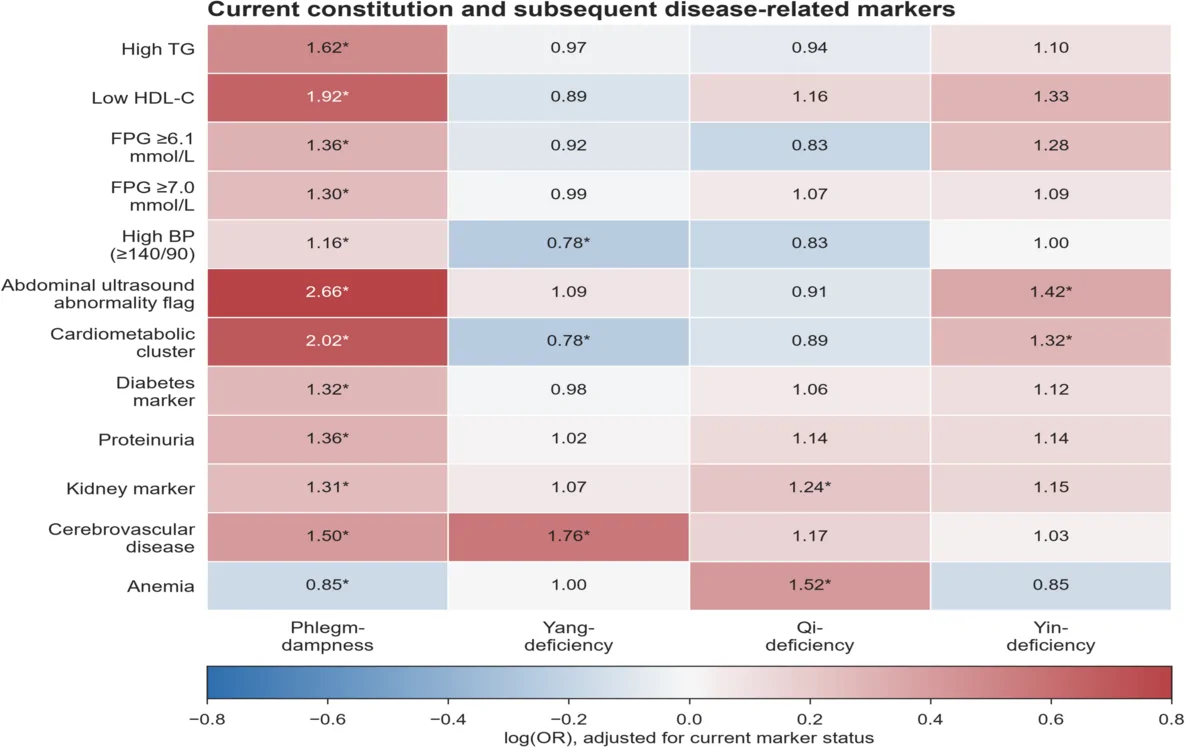
Current constitution and subsequent disease-related markers. Models were adjusted for age, sex, follow-up interval, and current marker status. Cell labels show percentages or odds ratios; * indicates a false discovery rate of q<0.05. BP: blood pressure; FPG: fasting plasma glucose; HDL-C: high-density lipoprotein cholesterol; OR: odds ratio; TG: triglycerides.

In lagged annual analyses, current constitution was associated with marker status at the next visit after adjustment for the current status of the same marker, age, sex, examination year, and follow-up interval (Figure 3; Table 3). Compared with balanced constitution, current phlegm-dampness constitution was associated with a subsequent abdominal ultrasound abnormality flag (OR 2.66, 95% CI 2.46‐2.88), cardiometabolic risk clustering (OR 2.02, 95% CI 1.88‐2.17), diabetes-related markers (OR 1.32, 95% CI 1.20‐1.45), urine protein trace or positive (OR 1.36, 95% CI 1.26‐1.48), kidney impairment or proteinuria (OR 1.31, 95% CI 1.21‐1.41), and recorded cerebrovascular disease (OR 1.50, 95% CI 1.29‐1.75). These estimates describe associations with subsequent recorded status and do not establish new disease development.

**Table 3.** Current constitution and subsequent disease-related markers.^a^

Outcome	Phlegm-dampness	Yang-deficiency	Qi-deficiency	Yin-deficiency
High triglycerides	1.62 (1.51‐1.73)*	0.97 (0.85‐1.11)	0.94 (0.78‐1.13)	1.10 (0.91‐1.33)
Low HDL-C^b^	1.92 (1.64‐2.25)*	0.89 (0.63‐1.26)	1.16 (0.75‐1.79)	1.33 (0.81‐2.20)
FPG^c^ ≥6.1 mmol/L	1.36 (1.26‐1.46)*	0.92 (0.78‐1.07)	0.83 (0.66‐1.05)	1.28 (1.04‐1.57)
FPG ≥7.0 mmol/L	1.30 (1.19‐1.43)*	0.99 (0.81‐1.21)	1.07 (0.80‐1.43)	1.09 (0.81‐1.46)
High BP^d^ (≥140/90 mm Hg)	1.16 (1.08‐1.23)*	0.78 (0.70‐0.88)*	0.83 (0.70‐0.98)	1.00 (0.83‐1.20)
Abdominal ultrasound abnormality flag	2.66 (2.46‐2.88)*	1.09 (0.96‐1.23)	0.91 (0.76‐1.09)	1.42 (1.16‐1.75)*
Cardiometabolic risk cluster	2.02 (1.88‐2.17)*	0.78 (0.69‐0.88)*	0.89 (0.76‐1.05)	1.32 (1.11‐1.58)*
Diabetes-related marker	1.32 (1.20‐1.45)*	0.98 (0.81‐1.19)	1.06 (0.81‐1.39)	1.12 (0.84‐1.48)
Urine protein trace/positive	1.36 (1.26‐1.48)*	1.02 (0.86‐1.19)	1.14 (0.92‐1.40)	1.14 (0.89‐1.45)
Kidney impairment or proteinuria	1.31 (1.21‐1.41)*	1.07 (0.93‐1.22)	1.24 (1.05‐1.48)*	1.15 (0.94‐1.42)
Recorded cerebrovascular disease	1.50 (1.29‐1.75)*	1.76 (1.40‐2.22)*	1.17 (0.81‐1.69)	1.03 (0.62‐1.72)
Anemia	0.85 (0.76‐0.94)*	1.00 (0.85‐1.17)	1.52 (1.23‐1.88)*	0.85 (0.65‐1.11)
eGFR^e^ <60	1.04 (0.91‐1.20)	1.23 (1.00‐1.51)	1.60 (1.24‐2.06)*	1.20 (0.87‐1.65)

a Values are odds ratios (95% CIs) adjusted for age, sex, examination year, follow-up interval, and current status of the same marker. * indicates a false discovery rate-adjusted q value <0.05.

b HDL-C: high-density lipoprotein cholesterol.

c FPG: fasting plasma glucose.

d BP: blood pressure.

e eGFR: estimated glomerular filtration rate.

Among biochemical risk markers, current phlegm-dampness was associated with subsequent high triglycerides (OR 1.62, 95% CI 1.51‐1.73), low HDL-C (OR 1.92, 95% CI 1.64‐2.25), fasting glucose ≥6.1 mmol/L (OR 1.36, 95% CI 1.26‐1.46), fasting glucose ≥7.0 mmol/L (OR 1.30, 95% CI 1.19‐1.43), any dyslipidemia (OR 1.20, 95% CI 1.12‐1.29), and high blood pressure (OR 1.16, 95% CI 1.08‐1.23).

Additional adiposity adjustment materially changed interpretation. After BMI adjustment, associations remained for a subsequent abdominal ultrasound abnormality flag (OR 1.46, 95% CI 1.33‐1.61) and cardiometabolic risk clustering (OR 1.19, 95% CI 1.09‐1.30). In contrast, associations with diabetes-related markers (OR 1.04, 95% CI 0.92‐1.18), proteinuria (OR 1.09, 95% CI 0.98‐1.20), kidney impairment or proteinuria (OR 1.07, 95% CI 0.97‐1.17), any dyslipidemia (OR 1.07, 95% CI 0.98‐1.17), and fasting glucose ≥6.1 mmol/L (OR 1.04, 95% CI 0.94‐1.15) were attenuated to include the null. New-onset analyses showed the same pattern: BMI-adjusted associations persisted for a new abdominal ultrasound abnormality flag (OR 1.43, 95% CI 1.24‐1.66) and new cardiometabolic risk clustering (OR 1.21, 95% CI 1.06‐1.38), whereas the other reviewed outcomes did not remain statistically distinguishable from the null. Waist circumference–adjusted models produced similar conclusions. Persistence analyses are reported separately from new-onset analyses in Multimedia Appendix 1.

Other constitution-specific longitudinal patterns were also observed. Yang-deficiency constitution was associated with subsequent recorded cerebrovascular disease (OR 1.76, 95% CI 1.40‐2.22) but was inversely associated with subsequent cardiometabolic risk clustering (OR 0.78, 95% CI 0.69‐0.88). Qi-deficiency constitution was associated with subsequent anemia (OR 1.52, 95% CI 1.23‐1.88) and eGFR <60 mL/min/1.73 m^2^ (OR 1.60, 95% CI 1.24‐2.06).

E-value analysis was used as a quantitative sensitivity analysis for selected longitudinal associations. The strongest robustness signals were observed for associations of phlegm-dampness constitution with a subsequent abdominal ultrasound abnormality flag (OR 2.66, 95% CI 2.46‐2.88; E-value for the confidence limit closest to the null=4.36), cardiometabolic risk clustering (OR 2.02, 95% CI 1.88‐2.17; E-value=3.17), low HDL-C (OR 1.92, 95% CI 1.64‐2.25; E-value=2.66), and high triglycerides (OR 1.62, 95% CI 1.51‐1.73; E-value=2.38). For the association between yang-deficiency constitution and subsequent recorded cerebrovascular disease, the corresponding E-value was 2.15. These sensitivity estimates do not establish causality but quantify how strong an unmeasured confounder would need to be to fully explain away each observed association.

### Constitution State Transitions

**Figure 4.**
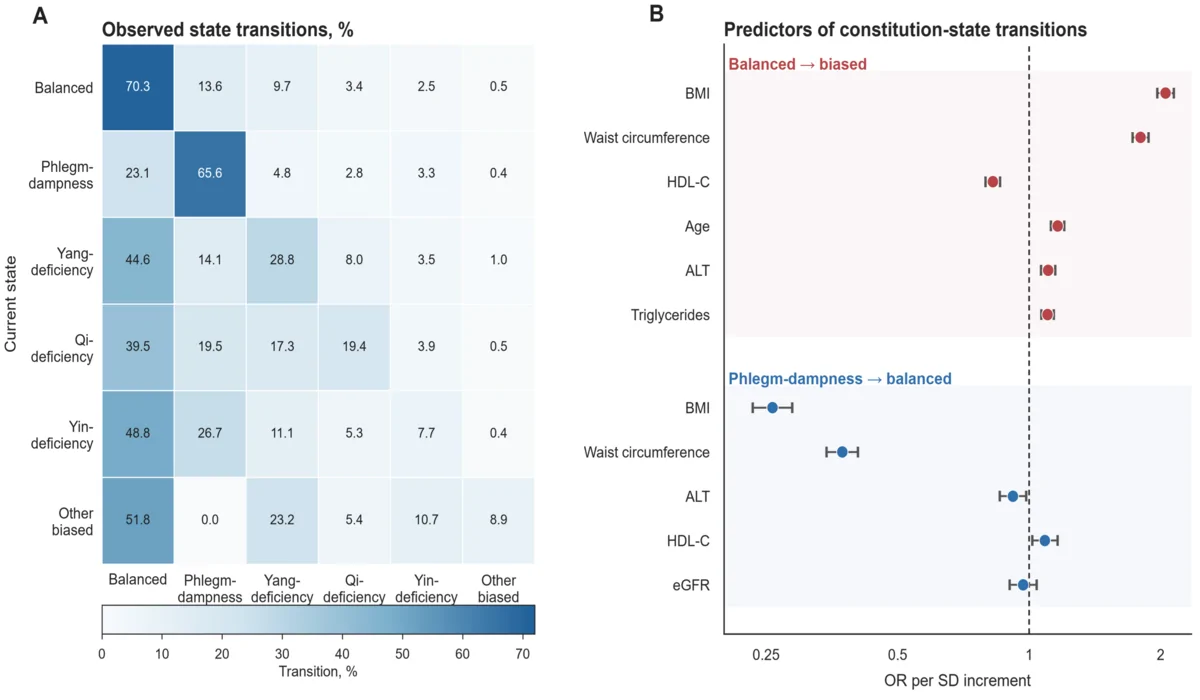
Constitution-state transition dynamics. (A) The adjacent state transition matrix; (B) presents standardized predictors of selected transitions. ALT: alanine aminotransferase; eGFR: estimated glomerular filtration rate; HDL-C: high-density lipoprotein cholesterol; OR: odds ratio.

Adjacent constitution state transitions showed that constitution was not a fixed label (Figure 4, panel A). Across adjacent observed records, the crude constitution group change rate was 35.6% (12833/36057). Among records initially classified as balanced constitution, 70.3% (17181/24424) remained balanced at the next observed visit, while 13.6% (3322/24424) transitioned to phlegm-dampness, 9.7% (2364/24424) to yang-deficiency, 3.4% (826/24424) to qi-deficiency, and 2.5% (601/24424) to yin-deficiency. Among records initially classified as phlegm-dampness, 65.6% (5260/8019) remained phlegm-dampness, and 23.1% (1850/8019) transitioned to balanced constitution.

Baseline adiposity and metabolic markers were associated with subsequent constitution state transitions (Figure 4, panel B). Among individuals with balanced constitution, higher BMI (OR 2.05 per SD, 95% CI 1.96‐2.14), larger waist circumference (OR 1.80 per SD, 95% CI 1.72‐1.88), older age (OR 1.16 per SD, 95% CI 1.12‐1.20), higher triglycerides (OR 1.10 per SD, 95% CI 1.07‐1.14), higher ALT (OR 1.11 per SD, 95% CI 1.06‐1.15), and higher fasting glucose (OR 1.06 per SD, 95% CI 1.03‐1.10) were associated with transition to biased constitution, whereas higher HDL-C was protective (OR 0.83 per SD, 95% CI 0.79‐0.86). Among individuals with phlegm-dampness constitution, higher BMI (OR 0.26 per SD, 95% CI 0.23‐0.29) and larger waist circumference (OR 0.37 per SD, 95% CI 0.34‐0.41) were associated with lower odds of returning to balanced constitution.

### Digital Constitution Prediction, Explainability, Calibration, and Utility

Finally, we evaluated whether routine examination variables could identify recorded constitution labels without using the 33 assessment items. In the original record-level temporal evaluation, XGBoost achieved an AUC of 0.935 and PR-AUC of 0.898 for phlegm-dampness label presence, while the broader biased-vs-balanced task showed moderate discrimination (XGBoost AUC, 0.733). However, 6694 participants overlapped between training and validation, 6382 between training and testing, and 6558 between validation and testing; 8122 of 11,355 participants appeared in at least 2 periods. We therefore interpret these results as future record prediction within a recurring cohort rather than as independent temporal validation.

In the participant-disjoint temporal sensitivity analysis, the training, validation, and test sets contained 9219, 1081, and 1054 participants, respectively. For phlegm-dampness label presence, full-model XGBoost achieved an AUC of 0.927 (95% CI 0.912‐0.941), a PR-AUC of 0.881, and a Brier score of 0.123. Full logistic regression achieved an AUC of 0.924 (95% CI 0.909‐0.938). A BMI-only logistic model achieved an AUC of 0.923 (95% CI 0.907‐0.938), and adding waist circumference did not improve discrimination (AUC 0.918, 95% CI 0.903‐0.933). Without anthropometric predictors, XGBoost AUC decreased to 0.685 (95% CI 0.653‐0.717). The corresponding participant-disjoint biased-vs-balanced XGBoost AUC was 0.739 (95% CI 0.708‐0.768). These results show that participant overlap did not explain the high phlegm-dampness discrimination, but BMI alone captured nearly all of that discrimination. The results of participant-disjoint temporal evaluation are summarized in Table 4.

**Table 4.** Participant-disjoint temporal evaluation of constitution label prediction models.^a^

Task, feature set, and model	AUC^b^ (95% CI)	PR-AUC^c^	Brier score	Test N	Test events
Biased vs balanced constitution					
BMI only					
Logistic regression	0.699 (0.665‐0.731)	0.896	0.290	1054	814
BMI and waist					
Logistic regression	0.714 (0.683‐0.746)	0.905	0.276	1054	814
Full clinical					
Logistic regression	0.734 (0.704‐0.764)	0.913	0.248	1054	814
XGBoost^d^	0.739 (0.708‐0.768)	0.912	0.318	1054	814
No anthropometry					
Logistic regression	0.608 (0.571‐0.648)	0.825	0.207	1054	814
XGBoost	0.608 (0.569‐0.650)	0.823	0.236	1054	814
Phlegm-dampness label vs others					
BMI only					
Logistic regression	0.923 (0.907‐0.938)	0.859	0.118	1054	376
BMI and waist					
Logistic regression	0.918 (0.903‐0.933)	0.864	0.122	1054	376
Full clinical					
Logistic regression	0.924 (0.909‐0.938)	0.874	0.111	1054	376
XGBoost	0.927 (0.912‐0.941)	0.881	0.123	1054	376
No anthropometry					
Logistic regression	0.681 (0.650‐0.711)	0.500	0.264	1054	376
XGBoost	0.685 (0.653‐0.717)	0.527	0.238	1054	376

a No participant appeared in more than 1 split.

b AUC: area under the receiver operating characteristic curve.

c PR-AUC: area under the precision-recall curve.

d XGBoost: extreme gradient boosting.

**Figure 5.**
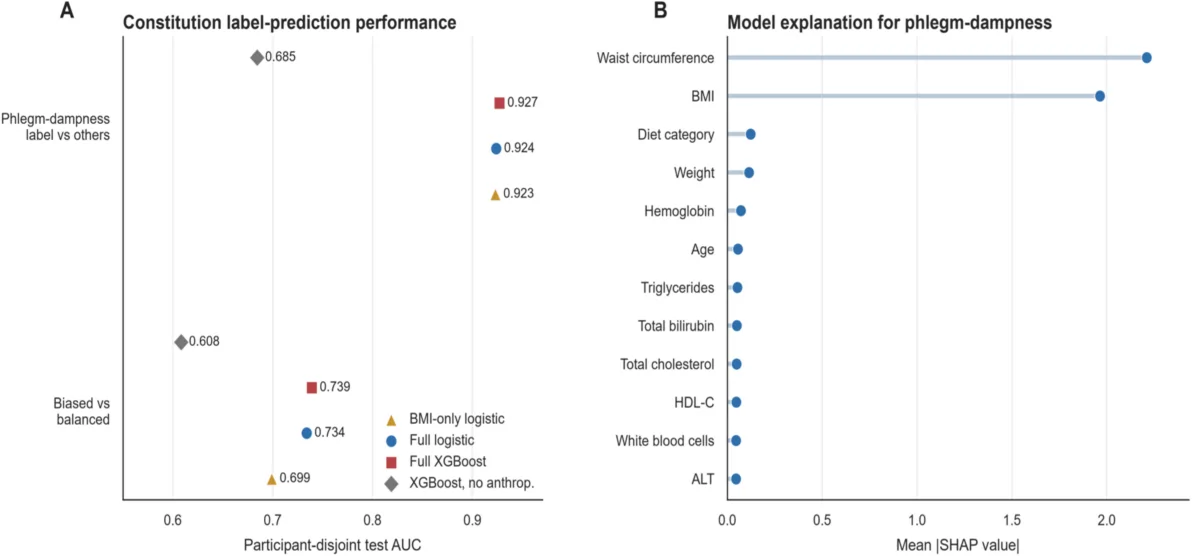
Constitution label prediction and model explanation. (A) Participant-disjoint temporal evaluation; (B) presents mean absolute Shapley additive explanations (SHAP) values for the original temporal extreme gradient boosting (XGBoost) phlegm-dampness label prediction model and excludes traditional Chinese medicine (TCM) assessment items. ALT: alanine aminotransferase; anthrop.: anthropometry; AUC: area under the receiver operating characteristic curve; HDL-C: high-density lipoprotein cholesterol.

SHAP-based interpretation of the original XGBoost phlegm-dampness model identified waist circumference and BMI as the dominant predictors, followed by body weight, hemoglobin, age, triglycerides, total bilirubin, total cholesterol, HDL-C, white blood cell count, ALT, creatinine, LDL-C, and diastolic blood pressure (Figure 5, panel B). Together with the BMI-only and no-anthropometry comparisons, this indicates that the model primarily reproduces an adiposity-centered component of the recorded phlegm-dampness label rather than a comprehensive constitution construct.

Calibration analyses showed that the phlegm-dampness prediction task was more stable than the broad biased-vs-balanced task under temporal validation (Multimedia Appendix 2). For phlegm-dampness prediction, the XGBoost model had a temporal test Brier score of 0.120, 10-bin expected calibration error of 0.085, and calibration slope of 0.65; the logistic regression model had a Brier score of 0.118, expected calibration error of 0.073, and calibration slope of 0.78. By contrast, the broad biased-vs-balanced task showed weaker calibration, reflecting temporal distribution shift in the 2025‐2026 test period.

Decision curve analysis suggested potential utility for the phlegm-dampness model across clinically plausible threshold probabilities. For the XGBoost phlegm-dampness model, net benefit exceeded both treat-all and treat-none strategies from threshold probabilities of 0.20 to 0.50, with a net benefit of 0.301 at a threshold of 0.20, 0.277 at a threshold of 0.30, 0.251 at a threshold of 0.40, and 0.228 at a threshold of 0.50. The broad biased-vs-balanced model showed less favorable decision curve behavior because of temporal changes in event prevalence.

Exploratory split-conformal summaries further highlighted the temporal stress imposed by the 2025‐2026 test period. For the XGBoost phlegm-dampness model, the nominal 90% set prediction procedure achieved empirical coverage of 0.83 with an average set size of 0.98 in the temporal test set. For the broader biased-vs-balanced task, empirical coverage was 0.70 with an average set size of 1.29. These results are best interpreted as exploratory uncertainty quantification under temporal shift rather than guaranteed clinical coverage. Supplementary tables for disease-to-constitution enrichment, burden score definitions, burden models, E-value sensitivity analysis, subgroup and interaction analyses, prediction sensitivity analysis, conformal uncertainty, variable missingness, and disease marker missingness are provided in Multimedia Appendix 3.

## Discussion

### Principal Findings

In this longitudinal analysis of 47,417 examination-constitution records of 11,355 older adults, we found that TCM constitution labels could be characterized as record-based phenotypes using routine health examination data. Phlegm-dampness constitution showed the clearest and most reproducible signal. At baseline, it was characterized by higher BMI, larger waist circumference, more frequent obesity, higher blood pressure, higher fasting glucose, and higher triglycerides than balanced constitution. In multimarker burden models, phlegm-dampness constitution was associated with substantially higher cardiometabolic marker burden and higher corrected, nonoverlapping examination-derived marker burden. In lagged annual models, current phlegm-dampness was associated with multiple subsequent marker states after adjustment for the current status of the same marker and other covariates; however, most glucose, lipid, kidney, and proteinuria associations attenuated after adiposity adjustment.

The findings also showed that constitution is dynamic rather than a fixed label. More than one-third of adjacent annual visit pairs changed constitution group, and adiposity-related markers were strongly associated with transitions. Among participants currently classified as balanced constitution, higher BMI and larger waist circumference were associated with transition to biased constitution. Among participants with phlegm-dampness constitution, higher BMI and larger waist circumference were associated with lower odds of returning to balanced constitution. These results support a longitudinal interpretation of constitution as a modifiable health state phenotype linked to body composition and metabolic status.

The label prediction analysis provided a complementary perspective. High discrimination persisted after enforcing complete participant separation, showing that overlap did not account for model performance. However, BMI alone nearly matched the full model, and performance fell markedly without anthropometric variables. The evidence therefore supports a reproducible adiposity-centered phlegm-dampness label phenotype and not a broad classifier that captures the complete TCM constitution construct.

### Contribution to Clinical Informatics

A central contribution of this study is methodological. We did not attempt to prove that a traditional taxonomy is equivalent to biomedical disease categories. Instead, we asked which measurable components of an existing culturally embedded label could be recovered from routine records, how those labels changed over time, and where the limits of prediction became visible. This framing preserves the local classification while subjecting it to reproducible data-driven characterization and explicit boundary testing.

This approach extends prior electronic health record phenotyping work, which has often focused on deriving disease labels from structured and unstructured clinical data [1]. It also aligns with efforts to make phenotype algorithms portable and transparent across clinical datasets [3]. Here, the target was not a conventional disease diagnosis but a health state taxonomy used in preventive care. The same logic could be applied to other culturally specific, functional, behavioral, or patient-centered classifications. The key requirement is not that the taxonomy originate from biomedicine but that it can be linked transparently to observable data, longitudinal outcomes, and uncertainty-aware prediction.

### Interpretation of Phlegm-Dampness as a Cardiometabolic Digital Phenotype

Among the constitution groups, phlegm-dampness had the strongest record-based signature. Its association with obesity, waist circumference, dyslipidemia, glucose abnormalities, and abdominal ultrasound flags is consistent with previous studies linking phlegm-dampness constitution with metabolic syndrome and cardiometabolic risk [10]. This anthropometric signal is partly expected because the standardized older adult scale directly incorporates BMI and waist-circumference categories into the phlegm-dampness score [16]. Therefore, the high AUC of a model containing those same measurements cannot be interpreted as independent validation of the full phlegm-dampness construct. The additional sensitivity analyses substantially narrowed interpretation: BMI alone produced almost the same discrimination as the full model, whereas model performance fell markedly after anthropometric variables were removed. Adiposity adjustment also explained most longitudinal associations with glucose, lipids, and kidney-related markers. Associations with subsequent abdominal ultrasound abnormality and cardiometabolic risk clustering persisted but were attenuated. Thus, the evidence identifies an adiposity-centered recorded phenotype with limited additional cardiometabolic information rather than a general digital representation of phlegm-dampness.

This distinction is important for interpreting the contribution of the prediction models. The close performance of the BMI-only model indicates that the strongest recoverable signal for the recorded phlegm-dampness label was largely captured by a simple anthropometric measure, whereas the decline after removing anthropometric variables indicates that body-size information was central to model performance. This finding does not invalidate the broader TCM constitution construct. Rather, it defines the information content of the operational phenotype that could be reconstructed from this routine examination dataset. The study therefore contributes not only a predictive framework but also an empirical assessment of construct boundaries and incremental predictive value. In practical terms, the recorded phenotype may support risk communication and hypothesis generation, but it should not be interpreted as an independent diagnostic classifier or as evidence that phlegm-dampness has a causal effect on metabolic disease.

These findings suggest that phlegm-dampness may function as an adiposity-centered cardiometabolic phenotype within this older adult examination cohort. However, this interpretation should remain descriptive rather than causal. The constitution label and several risk markers were measured in routine practice rather than under a prospective experimental protocol. The observed associations may reflect shared upstream determinants, including body composition, diet, activity, socioeconomic factors, medication use, comorbidities, and health care use. E-value analyses quantified the strength of unmeasured confounding that would be required to explain selected longitudinal associations, but they do not eliminate the possibility of residual confounding [23].

### Why Bidirectional Mapping Matters for Patients and Clinicians

The bidirectional analysis addresses a practical communication gap. Patients may ask 2 different questions: “Given my disease or examination abnormality, which constitution pattern is common?” and “Given my constitution, which health problems should I pay attention to?” These questions are not statistically identical. Disease-to-constitution mapping showed which constitution patterns were overrepresented among people with specific recorded diseases or examination-derived markers. Constitution-to-disease modeling estimated the adjusted association between a constitution state and each marker relative to balanced constitution.

This bidirectional framing may make constitution research more interpretable to clinicians and patients outside TCM. It also reduces the risk of presenting constitution as a single-direction causal exposure. For clinical informatics applications, such mapping could support patient-facing explanations, preventive counseling, and hypothesis generation. For example, a decision support interface could use the disease-to-constitution map to contextualize a patient’s abnormal ultrasound or metabolic markers, while using the constitution-to-risk map to highlight follow-up priorities. Such applications would require prospective evaluation before clinical use.

### Model Evaluation Beyond AUC

The phlegm-dampness label prediction model achieved high temporal test discrimination, but the broader evaluation showed why AUC alone is insufficient. Calibration analyses suggested that model probabilities were not perfectly calibrated under temporal shift, and the broad biased-vs-balanced model showed weaker calibration than the phlegm-dampness model. Decision curve analysis suggested potential net benefit for phlegm-dampness label prediction across the evaluated threshold probabilities, but this retrospective analysis does not establish clinical effectiveness. Exploratory conformal summaries further demonstrated that uncertainty behavior differed across tasks when the model was evaluated in a later calendar period.

These findings align with broader concerns in clinical prediction and artificial intelligence evaluation. Prediction models can appear accurate in one setting yet perform differently when prevalence, measurement practices, population characteristics, or care pathways change [24]. Transparent reporting is therefore essential when prediction models are developed from routine clinical data [17]. For clinical informatics tools, calibration is especially important because users may interpret model outputs as actionable probabilities rather than simple risk rankings [12]. Decision curve analysis provides a complementary view of potential clinical utility, but it cannot replace prospective implementation evaluation [13]. Our results therefore support the feasibility of digital constitution phenotyping while emphasizing that deployment would require prospective validation, workflow integration, and monitoring for temporal drift.

### Privacy-Preserving Reproducibility and Deployment Boundary

This study illustrates a common tension in clinical informatics research: the data most relevant for phenotyping often cannot be fully open because they contain sensitive health information. Algorithmic governance discussions have emphasized that transparency must be balanced with privacy, accountability, and contextual risk [25]. We addressed this tension by separating reproducible code and aggregate evidence from restricted participant-level data. The public package contains scripts, data dictionaries, aggregate output tables, figure-generation workflows, a file manifest, and synthetic demonstration data, while the original workbook and individual-level derived analysis frames remain restricted. This approach is consistent with the findable, accessible, interoperable, reusable (FAIR) data management principles in that reusable metadata and analytic outputs are shared even when individual-level data cannot be open [26].

The same privacy-aware framing is important for implementation. Adaptive mobile health design work has shown that patient-facing systems require explicit workflows, adaptation logic, usability assessment, and carefully defined deployment boundaries rather than a prediction model alone [27]. Our models are therefore best viewed as research prototypes for decision support and communication. They should not replace clinician assessment or the original TCM constitution assessment, and any patient-facing use would require prospective workflow evaluation, local recalibration, and clear explanation of uncertainty.

### Strengths

This study has several strengths. It used a large longitudinal routine examination dataset with repeated constitution assessments, separated recorded diseases from examination-derived marker flags, used bidirectional mapping, and evaluated constitution state transitions. The additional analyses also quantified participant overlap, added a strictly participant-disjoint temporal evaluation, compared the full model against simple adiposity baselines, separated new onset from persistence, adjusted longitudinal associations for adiposity, and corrected overlapping burden components. These additions make both performance and limitations more transparent.

### Limitations

Several limitations should be considered. First, this observational study cannot establish causal effects of constitution on disease. Adjustment for current marker status does not eliminate residual confounding from disease severity, treatment, repeated measurements, or health care use. Second, the standardized older adult scale explicitly includes BMI and waist circumference in phlegm-dampness scoring. Therefore, strong performance of anthropometry-based models partly reflects criterion overlap rather than independent recovery of constitution from unrelated biomarkers. Although the system automatically calculated scores and the resulting labels were confirmed by a clinician, the analytic export did not preserve assessor identity or a detailed correction audit trail. We could not quantify how often preliminary classifications changed during confirmation or evaluate between-assessor variation. Third, participants were residents of 2 communities in Deyang City undergoing routine health examinations; generalizability to other regions, recruitment settings, and age groups requires external validation. Fourth, ECG and abdominal ultrasound were available only as heterogeneous structured normal or abnormal flags; specific findings and free-text interpretations were unavailable. Fifth, medication names were recorded in only 42.3% of records, and blanks could not be distinguished from no medication use, preventing reliable adjustment. Socioeconomic status, detailed lifestyle intensity, and adjudicated diagnoses were also unavailable. Sixth, the original calendar-year evaluation included repeated participants across periods. Participant-disjoint sensitivity analysis reduced this bias but remains an internal validation within the same source cohort. Seventh, BMI alone nearly matched the full phlegm-dampness model, and most longitudinal cardiometabolic associations attenuated after adiposity adjustment. Because BMI, waist circumference, and the constitution label share information by design, residual construct overlap and collinearity are unavoidable in models that include anthropometry. Accordingly, adiposity adjustment in this study evaluates statistical incremental value rather than causal mediation or equivalence between phlegm-dampness constitution and adiposity. The model should therefore not be interpreted as a comprehensive constitution classifier. Eighth, the other biased constitutions group was too small for stable inference. Finally, recurrence was not evaluated, and subgroup analyses did not constitute a full fairness audit.

### Future Work

Future studies should validate this digital phenotyping framework in independent cohorts, younger age groups, different regions, and other clinical and health examination settings. Prospective studies are needed to determine whether digital constitution phenotyping improves preventive counseling, risk communication, or follow-up adherence. Model development should incorporate external validation, calibration updating, subgroup performance assessment, fairness analysis, and human-centered evaluation, consistent with broader recommendations for health equity in machine learning [28]. If digital constitution tools are deployed, they should be presented as decision support and communication aids rather than stand-alone diagnostic systems. Future research should also examine whether longitudinal changes in body composition, lifestyle, medication use, and metabolic markers precede constitution transitions or whether constitution changes mainly reflect concurrent health state reassessment.

### Conclusions

Routine health examination data captured a reproducible, predominantly adiposity-centered phlegm-dampness label phenotype in this older adult cohort. Strong discrimination persisted in participant-disjoint evaluation but was nearly matched by BMI alone, and several longitudinal associations were explained by adiposity. The framework is useful for transparent characterization of an existing culturally embedded health state label, but it does not establish a stand-alone diagnostic system or a comprehensive digital classifier of TCM constitution.

## Supplementary material

10.2196/100063 Multimedia Appendix 1 Additional sensitivity analyses.

10.2196/100063 Multimedia Appendix 2 Temporal test calibration curves for digital constitution screening models. Calibration curves compare mean predicted probability with observed event rate across deciles of predicted risk in the 2025-2026 temporal test set. (A) Biased vs balanced constitution and (B) phlegm-dampness label presence vs all other records. The dashed diagonal line indicates ideal calibration.

10.2196/100063 Multimedia Appendix 3 Supplementary tables for disease-to-constitution enrichment, burden score definitions, burden models, E-value sensitivity analysis, subgroup and interaction analyses, prediction sensitivity analysis, conformal uncertainty, variable missingness, and disease marker missingness.

## Abbreviations

ALT: alanine aminotransferase
AST: aspartate aminotransferase
AUC: area under the receiver operating characteristic curve
ECG: electrocardiogram
eGFR: estimated glomerular filtration rate
FAIR: findable, accessible, interoperable, reusable
HDL-C: high-density lipoprotein cholesterol
IRR: incidence rate ratio
LDL-C: low-density lipoprotein cholesterol
OR: odds ratio
PR-AUC: precision-recall area under the curve
PROBAST: Prediction Model Risk of Bias Assessment Tool
RECORD: Reporting of Studies Conducted Using Observational Routinely Collected Health Data
SHAP: Shapley additive explanations
STROBE: Strengthening the Reporting of Observational Studies in Epidemiology
SVG: scalable vector graphics
TCM: traditional Chinese medicine
TRIPOD: Transparent Reporting of a Multivariable Prediction Model for Individual Prognosis or Diagnosis
XGBoost: extreme gradient boosting
