# Supplementary material for: Longitudinal Digital Phenotyping of Traditional Chinese Medicine Constitution From Routine Health Examination Records: Retrospective Clinical Informatics Study
Source: JMIR Med Inform. 2026 Sep 23;14:e100063. doi: 10.2196/100063 (PMC13600657; doi:10.2196/100063)
Supplement: Supplementary file 1 [file medinform-v14-e100063-s001.docx]

Multimedia Appendix 12. Additional sensitivity analyses.

This appendix reports additional sensitivity analyses of participant overlap, participant-disjoint temporal label prediction, adiposity baselines, adiposity-adjusted subsequent-status models, new-onset and persistence outcomes, corrected nonoverlapping burden components, and medication-field completeness.

## *Participant overlap across the original calendar-year periods*

| comparison | participants |
| --- | --- |
| Training 2017-2023 | 9220 |
| Validation 2024 | 7775 |
| Test 2025-2026 | 8238 |
| Overlap: Training 2017-2023 and Validation 2024 | 6694 |
| Overlap: Training 2017-2023 and Test 2025-2026 | 6382 |
| Overlap: Validation 2024 and Test 2025-2026 | 6558 |
| Overlap across all three temporal splits | 5756 |
| Participants appearing in at least two temporal splits | 8122 |

## *Participant-disjoint temporal label-prediction performance*

| Task | Feature set | Model | AUC (95% CI) | PR-AUC | Brier score | Test n | Test events |
| --- | --- | --- | --- | --- | --- | --- | --- |
| Biased vs balanced constitution | BMI only | Logistic regression | 0.699 (0.665-0.731) | 0.896 | 0.290 | 1,054 | 814 |
| Biased vs balanced constitution | BMI and waist | Logistic regression | 0.714 (0.683-0.746) | 0.905 | 0.276 | 1,054 | 814 |
| Biased vs balanced constitution | Full clinical | Logistic regression | 0.734 (0.704-0.764) | 0.913 | 0.248 | 1,054 | 814 |
| Biased vs balanced constitution | Full clinical | XGBoost | 0.739 (0.708-0.768) | 0.912 | 0.318 | 1,054 | 814 |
| Biased vs balanced constitution | No anthropometry | Logistic regression | 0.608 (0.571-0.648) | 0.825 | 0.207 | 1,054 | 814 |
| Biased vs balanced constitution | No anthropometry | XGBoost | 0.608 (0.569-0.650) | 0.823 | 0.236 | 1,054 | 814 |
| Phlegm-dampness label vs others | BMI only | Logistic regression | 0.923 (0.907-0.938) | 0.859 | 0.118 | 1,054 | 376 |
| Phlegm-dampness label vs others | BMI and waist | Logistic regression | 0.918 (0.903-0.933) | 0.864 | 0.122 | 1,054 | 376 |
| Phlegm-dampness label vs others | Full clinical | Logistic regression | 0.924 (0.909-0.938) | 0.874 | 0.111 | 1,054 | 376 |
| Phlegm-dampness label vs others | Full clinical | XGBoost | 0.927 (0.912-0.941) | 0.881 | 0.123 | 1,054 | 376 |
| Phlegm-dampness label vs others | No anthropometry | Logistic regression | 0.681 (0.650-0.711) | 0.500 | 0.264 | 1,054 | 376 |
| Phlegm-dampness label vs others | No anthropometry | XGBoost | 0.685 (0.653-0.717) | 0.527 | 0.238 | 1,054 | 376 |

## *Subsequent marker status conditional on current status*

| Outcome | Adjustment | n | Events | OR (95% CI) | P value |
| --- | --- | --- | --- | --- | --- |
| Abnormal abdominal ultrasound flag | Base | 27,275 | 17,398 | 2.66 (2.46-2.88) | <.001 |
| Abnormal abdominal ultrasound flag | BMI | 27,267 | 17,394 | 1.46 (1.33-1.61) | <.001 |
| Abnormal abdominal ultrasound flag | Waist | 27,270 | 17,394 | 1.44 (1.31-1.59) | <.001 |
| Abnormal abdominal ultrasound flag | BMI and waist | 27,263 | 17,391 | 1.30 (1.18-1.44) | <.001 |
| Cardiometabolic risk cluster | Base | 29,351 | 16,993 | 2.02 (1.88-2.17) | <.001 |
| Cardiometabolic risk cluster | BMI | 29,343 | 16,990 | 1.19 (1.09-1.30) | <.001 |
| Cardiometabolic risk cluster | Waist | 29,345 | 16,987 | 1.34 (1.23-1.45) | <.001 |
| Cardiometabolic risk cluster | BMI and waist | 29,338 | 16,985 | 1.15 (1.05-1.26) | 0.002 |
| Diabetes-related marker | Base | 29,197 | 5,551 | 1.32 (1.20-1.45) | <.001 |
| Diabetes-related marker | BMI | 29,189 | 5,550 | 1.04 (0.92-1.18) | 0.529 |
| Diabetes-related marker | Waist | 29,191 | 5,549 | 0.99 (0.88-1.12) | 0.933 |
| Diabetes-related marker | BMI and waist | 29,184 | 5,549 | 0.97 (0.86-1.10) | 0.654 |
| Proteinuria trace/positive | Base | 28,355 | 4,626 | 1.36 (1.26-1.48) | <.001 |
| Proteinuria trace/positive | BMI | 28,347 | 4,625 | 1.09 (0.98-1.20) | 0.112 |
| Proteinuria trace/positive | Waist | 28,349 | 4,625 | 1.08 (0.98-1.20) | 0.127 |
| Proteinuria trace/positive | BMI and waist | 28,342 | 4,625 | 1.04 (0.94-1.16) | 0.458 |
| Kidney impairment or proteinuria | Base | 29,157 | 6,088 | 1.31 (1.21-1.41) | <.001 |
| Kidney impairment or proteinuria | BMI | 29,149 | 6,087 | 1.07 (0.97-1.17) | 0.184 |
| Kidney impairment or proteinuria | Waist | 29,151 | 6,087 | 1.05 (0.95-1.15) | 0.321 |
| Kidney impairment or proteinuria | BMI and waist | 29,144 | 6,087 | 1.02 (0.92-1.12) | 0.716 |
| Any dyslipidemia | Base | 29,123 | 18,800 | 1.20 (1.12-1.29) | <.001 |
| Any dyslipidemia | BMI | 29,115 | 18,796 | 1.07 (0.98-1.17) | 0.112 |
| Any dyslipidemia | Waist | 29,117 | 18,794 | 1.04 (0.96-1.14) | 0.349 |
| Any dyslipidemia | BMI and waist | 29,110 | 18,791 | 1.03 (0.95-1.13) | 0.465 |
| FPG ≥6.1 mmol/L | Base | 29,158 | 8,874 | 1.36 (1.26-1.46) | <.001 |
| FPG ≥6.1 mmol/L | BMI | 29,150 | 8,873 | 1.04 (0.94-1.15) | 0.438 |
| FPG ≥6.1 mmol/L | Waist | 29,152 | 8,870 | 0.97 (0.88-1.07) | 0.508 |
| FPG ≥6.1 mmol/L | BMI and waist | 29,145 | 8,870 | 0.95 (0.86-1.05) | 0.314 |
| High triglycerides | Base | 29,112 | 9,059 | 1.62 (1.51-1.73) | <.001 |
| High triglycerides | BMI | 29,104 | 9,059 | 1.11 (1.01-1.21) | 0.023 |
| High triglycerides | Waist | 29,106 | 9,054 | 1.02 (0.94-1.11) | 0.637 |
| High triglycerides | BMI and waist | 29,099 | 9,054 | 0.98 (0.90-1.07) | 0.658 |
| Low HDL-C | Base | 28,019 | 934 | 1.92 (1.64-2.25) | <.001 |
| Low HDL-C | BMI | 28,011 | 934 | 1.26 (1.03-1.55) | 0.028 |
| Low HDL-C | Waist | 28,014 | 934 | 1.11 (0.91-1.37) | 0.308 |
| Low HDL-C | BMI and waist | 28,007 | 934 | 1.08 (0.88-1.34) | 0.458 |

## *New onset among participants negative at the current visit*

| Outcome | Adjustment | n | Events | OR (95% CI) | P value |
| --- | --- | --- | --- | --- | --- |
| Abnormal abdominal ultrasound flag | Base | 10,596 | 3,479 | 2.70 (2.38-3.06) | <.001 |
| Abnormal abdominal ultrasound flag | BMI | 10,590 | 3,476 | 1.43 (1.24-1.66) | <.001 |
| Abnormal abdominal ultrasound flag | Waist | 10,594 | 3,478 | 1.38 (1.19-1.59) | <.001 |
| Abnormal abdominal ultrasound flag | BMI and waist | 10,588 | 3,475 | 1.24 (1.06-1.44) | 0.006 |
| Cardiometabolic risk cluster | Base | 12,792 | 3,923 | 1.90 (1.69-2.14) | <.001 |
| Cardiometabolic risk cluster | BMI | 12,787 | 3,922 | 1.21 (1.06-1.38) | 0.005 |
| Cardiometabolic risk cluster | Waist | 12,792 | 3,923 | 1.29 (1.13-1.47) | <.001 |
| Cardiometabolic risk cluster | BMI and waist | 12,787 | 3,922 | 1.16 (1.02-1.33) | 0.027 |
| Diabetes-related marker | Base | 23,902 | 1,471 | 1.61 (1.42-1.82) | <.001 |
| Diabetes-related marker | BMI | 23,896 | 1,471 | 1.11 (0.94-1.31) | 0.207 |
| Diabetes-related marker | Waist | 23,899 | 1,471 | 1.08 (0.92-1.28) | 0.339 |
| Diabetes-related marker | BMI and waist | 23,893 | 1,471 | 1.02 (0.86-1.21) | 0.807 |
| Proteinuria trace/positive | Base | 23,843 | 2,501 | 1.41 (1.28-1.57) | <.001 |
| Proteinuria trace/positive | BMI | 23,836 | 2,501 | 1.10 (0.97-1.25) | 0.136 |
| Proteinuria trace/positive | Waist | 23,838 | 2,501 | 1.08 (0.95-1.23) | 0.220 |
| Proteinuria trace/positive | BMI and waist | 23,831 | 2,501 | 1.04 (0.91-1.19) | 0.551 |
| Kidney impairment or proteinuria | Base | 23,102 | 3,140 | 1.36 (1.23-1.50) | <.001 |
| Kidney impairment or proteinuria | BMI | 23,095 | 3,140 | 1.05 (0.93-1.19) | 0.400 |
| Kidney impairment or proteinuria | Waist | 23,098 | 3,140 | 1.05 (0.93-1.19) | 0.428 |
| Kidney impairment or proteinuria | BMI and waist | 23,091 | 3,140 | 1.00 (0.88-1.14) | 0.984 |
| Any dyslipidemia | Base | 10,522 | 3,513 | 1.22 (1.09-1.37) | <.001 |
| Any dyslipidemia | BMI | 10,517 | 3,511 | 1.07 (0.93-1.22) | 0.356 |
| Any dyslipidemia | Waist | 10,520 | 3,511 | 1.02 (0.89-1.17) | 0.771 |
| Any dyslipidemia | BMI and waist | 10,515 | 3,509 | 1.01 (0.88-1.17) | 0.841 |
| FPG ≥6.1 mmol/L | Base | 20,475 | 2,204 | 1.55 (1.39-1.73) | <.001 |
| FPG ≥6.1 mmol/L | BMI | 20,469 | 2,204 | 1.11 (0.97-1.28) | 0.123 |
| FPG ≥6.1 mmol/L | Waist | 20,473 | 2,204 | 1.11 (0.96-1.27) | 0.152 |
| FPG ≥6.1 mmol/L | BMI and waist | 20,467 | 2,204 | 1.05 (0.91-1.21) | 0.542 |
| High triglycerides | Base | 20,020 | 3,174 | 1.71 (1.55-1.89) | <.001 |
| High triglycerides | BMI | 20,013 | 3,174 | 1.05 (0.93-1.19) | 0.423 |
| High triglycerides | Waist | 20,016 | 3,171 | 0.99 (0.88-1.12) | 0.888 |
| High triglycerides | BMI and waist | 20,010 | 3,171 | 0.92 (0.81-1.05) | 0.231 |
| Low HDL-C | Base | 27,212 | 587 | 2.06 (1.71-2.47) | <.001 |
| Low HDL-C | BMI | 27,204 | 587 | 1.25 (0.98-1.59) | 0.066 |
| Low HDL-C | Waist | 27,207 | 587 | 1.08 (0.85-1.38) | 0.520 |
| Low HDL-C | BMI and waist | 27,200 | 587 | 1.05 (0.82-1.35) | 0.680 |

## *Persistence among participants positive at the current visit*

| Outcome | Adjustment | n | Events | OR (95% CI) | P value |
| --- | --- | --- | --- | --- | --- |
| Abnormal abdominal ultrasound flag | Base | 16,679 | 13,919 | 2.56 (2.30-2.86) | <.001 |
| Abnormal abdominal ultrasound flag | BMI | 16,677 | 13,918 | 1.50 (1.31-1.72) | <.001 |
| Abnormal abdominal ultrasound flag | Waist | 16,676 | 13,916 | 1.51 (1.32-1.73) | <.001 |
| Abnormal abdominal ultrasound flag | BMI and waist | 16,675 | 13,916 | 1.38 (1.20-1.58) | <.001 |
| Cardiometabolic risk cluster | Base | 16,559 | 13,070 | 2.10 (1.91-2.31) | <.001 |
| Cardiometabolic risk cluster | BMI | 16,556 | 13,068 | 1.17 (1.03-1.32) | 0.013 |
| Cardiometabolic risk cluster | Waist | 16,553 | 13,064 | 1.36 (1.21-1.52) | <.001 |
| Cardiometabolic risk cluster | BMI and waist | 16,551 | 13,063 | 1.13 (1.00-1.28) | 0.051 |
| Diabetes-related marker | Base | 5,295 | 4,080 | 0.99 (0.85-1.15) | 0.869 |
| Diabetes-related marker | BMI | 5,293 | 4,079 | 0.96 (0.79-1.17) | 0.691 |
| Diabetes-related marker | Waist | 5,292 | 4,078 | 0.89 (0.74-1.07) | 0.221 |
| Diabetes-related marker | BMI and waist | 5,291 | 4,078 | 0.92 (0.76-1.12) | 0.400 |
| Proteinuria trace/positive | Base | 4,512 | 2,125 | 1.27 (1.09-1.48) | 0.002 |
| Proteinuria trace/positive | BMI | 4,511 | 2,124 | 1.06 (0.88-1.26) | 0.559 |
| Proteinuria trace/positive | Waist | 4,511 | 2,124 | 1.08 (0.90-1.29) | 0.420 |
| Proteinuria trace/positive | BMI and waist | 4,511 | 2,124 | 1.04 (0.86-1.25) | 0.711 |
| Kidney impairment or proteinuria | Base | 6,055 | 2,948 | 1.24 (1.08-1.43) | 0.002 |
| Kidney impairment or proteinuria | BMI | 6,054 | 2,947 | 1.09 (0.92-1.29) | 0.305 |
| Kidney impairment or proteinuria | Waist | 6,053 | 2,947 | 1.08 (0.91-1.27) | 0.386 |
| Kidney impairment or proteinuria | BMI and waist | 6,053 | 2,947 | 1.06 (0.89-1.26) | 0.506 |
| Any dyslipidemia | Base | 18,601 | 15,287 | 1.20 (1.09-1.32) | <.001 |
| Any dyslipidemia | BMI | 18,598 | 15,285 | 1.08 (0.96-1.21) | 0.202 |
| Any dyslipidemia | Waist | 18,597 | 15,283 | 1.06 (0.95-1.19) | 0.306 |
| Any dyslipidemia | BMI and waist | 18,595 | 15,282 | 1.05 (0.93-1.18) | 0.423 |
| FPG ≥6.1 mmol/L | Base | 8,683 | 6,670 | 1.16 (1.03-1.31) | 0.017 |
| FPG ≥6.1 mmol/L | BMI | 8,681 | 6,669 | 0.97 (0.83-1.12) | 0.657 |
| FPG ≥6.1 mmol/L | Waist | 8,679 | 6,666 | 0.83 (0.71-0.97) | 0.016 |
| FPG ≥6.1 mmol/L | BMI and waist | 8,678 | 6,666 | 0.86 (0.73-1.00) | 0.054 |
| High triglycerides | Base | 9,092 | 5,885 | 1.50 (1.35-1.67) | <.001 |
| High triglycerides | BMI | 9,091 | 5,885 | 1.17 (1.02-1.33) | 0.020 |
| High triglycerides | Waist | 9,090 | 5,883 | 1.06 (0.93-1.21) | 0.349 |
| High triglycerides | BMI and waist | 9,089 | 5,883 | 1.05 (0.92-1.20) | 0.469 |
| Low HDL-C | Base | 807 | 347 | 1.62 (1.17-2.23) | 0.004 |
| Low HDL-C | BMI | 807 | 347 | 1.41 (0.93-2.14) | 0.103 |
| Low HDL-C | Waist | 807 | 347 | 1.36 (0.91-2.02) | 0.132 |
| Low HDL-C | BMI and waist | 807 | 347 | 1.33 (0.87-2.04) | 0.186 |

## *Corrected nonoverlapping examination-derived marker burden*

| burden | included_markers | revision_note |
| --- | --- | --- |
| Corrected examination-derived marker burden | High blood pressure; fasting glucose ≥7.0 mmol/L; any dyslipidemia; abnormal ECG flag; abnormal abdominal ultrasound flag; proteinuria; glycosuria; hematuria; liver enzyme elevation; anemia; eGFR <60 mL/min/1.73 m² | Proteinuria and kidney impairment were represented as separate nonoverlapping components; the composite kidney impairment or proteinuria variable was removed. |

| Constitution | Reference | n | IRR (95% CI) | P value |
| --- | --- | --- | --- | --- |
| Phlegm-dampness | Balanced | 10000 | 1.23 (1.20-1.25) | <.001 |
| Yang-deficiency | Balanced | 8484 | 1.05 (1.01-1.09) | 0.020 |
| Qi-deficiency | Balanced | 8101 | 1.18 (1.12-1.24) | <.001 |
| Yin-deficiency | Balanced | 8096 | 1.12 (1.05-1.19) | <.001 |

## *Medication-field completeness*

| Source field | Records | Nonmissing records | Completeness, % | Unique nonmissing values |
| --- | --- | --- | --- | --- |
| Medication name | 47417 | 20055 | 42.3 | 728 |
| Medication-use timing | 47417 | 18437 | 38.9 | 33 |
| Medication adherence | 47417 | 23105 | 48.7 | 3 |

Blank medication fields could not be distinguished from no medication use, and medication names were not standardized. Medication was therefore not included as a covariate; incomplete ascertainment remains a source of residual confounding.
